# Recent advances in understanding Crimean–Congo hemorrhagic fever virus

**DOI:** 10.12688/f1000research.16189.1

**Published:** 2018-10-29

**Authors:** David W. Hawman, Heinz Feldmann

**Affiliations:** 1 Laboratory of Virology, Division of Intramural Research, NIAID/NIH, Hamilton, Montana, 59840, USA

**Keywords:** Crimean-Congo hemorrhagic fever virus, CCHFV, CCHF disease, pathogenesis, animal models, vaccines, antivirals

## Abstract

Crimean-Congo hemorrhagic fever virus (CCHFV) is a widely distributed hemorrhagic fever virus and the cause of hemorrhagic disease in Africa, Southern and Eastern Europe, the Middle East, India and Asia. Recent emergence of CCHFV into Spain indicates that the geographic range of this virus is expanding and the presence of its tick vector in several countries without reported disease suggest that CCHFV will continue to spread. Research into CCHFV was historically limited by a lack of suitable animal models and tools to study viral pathogenesis. However, in the past few years the toolset for studying CCHFV has expanded with small animal and non-human primate models for CCHFV being developed along with a reverse genetics system that allows for investigation of viral determinants of disease. These tools have been utilized to understand how CCHFV antagonizes host restriction factors and to develop novel vaccine candidates that may help limit the substantial morbidity and mortality in humans caused by CCHFV.

## Introduction

**Figure 1.**
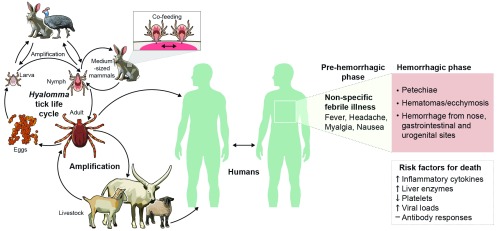
Crimean–Congo hemorrhagic fever virus (CCHFV). The natural reservoir and vector for CCHFV are ticks of the *Hyalomma* genus. Ticks can become infected at any life-cycle stage during feeding on a viremic animal or during co-feeding with an infected tick, and mammals likely act as important amplification hosts for the virus. Humans are at risk of CCHFV infection from tick bites, animal husbandry, and butchering of infected livestock and during the care of patients with CCHF. In humans, CCHF first presents as a non-specific febrile illness with a sudden onset of fever, headache, myalgia, and nausea. The disease can rapidly progress to the hemorrhagic phase of disease, during which patients exhibit petechiae, hematomas/ecchymosis, and hemorrhages from various sites around the body. Risk factors for death include elevated inflammatory cytokines and liver enzymes, high viral loads, decreased platelets, and absent antibody responses.

Crimean–Congo hemorrhagic fever virus (CCHFV) is a negative-sense RNA virus in the *Nairoviridae* family within the *Bunyavirales* order of viruses. CCHFV contains three genomic segments: small and medium, which encode for the nucleoprotein and glycoproteins, respectively, and a large segment encoding the RNA-dependent RNA-polymerase. CCHF as a disease was first described in humans in the 1940s when soldiers re-occupying abandoned farmland in the Crimea became ill with a hemorrhagic disease ^1^. In the late 1960s, it was discovered that the causative agent of this hemorrhagic disease in the Crimea was similar to the causative agent of hemorrhagic disease in the Belgian Congo (current Democratic Republic of the Congo) ^2^, and the name “Crimean–Congo hemorrhagic fever virus” was ascribed to the pathogen. The main vector and reservoir of CCHFV are hard-body ticks principally of the *Hyalomma* genus, although there is limited evidence that other species of ticks such as *Rhipicephalus* and *Dermacentor* species may be vectors ^3^. Vertebrate hosts such as domestic livestock and wild animals such as hares likely serve as amplifying hosts of CCHFV, with uninfected ticks becoming infected during feeding on viremic animals or during co-feeding with infected ticks ^4– 6^ ( Figure 1). The *Hyalomma* vector is found throughout Africa, Southern and Eastern Europe, the Middle East, India, and Asia and cases of CCHF are reported throughout these regions ^7^; an estimated 10,000 to 15,000 human infections with CCHFV occur each year, although most of these are subclinical and unrecognized ^7^. In correlation with the extensive geographic distribution of CCHFV, CCHFV exhibits substantial genetic diversity among geographically distinct isolates; isolates differ at the amino acid level by 5% in the nucleoprotein and L protein and up to 25% in the glycoprotein precursor ^3^.

## Disease and diagnosis

Humans can become infected with CCHFV via tick bites and butchering of infected livestock and in the health-care setting during the care of infected patients ^8^ ( Figure 1). Following an incubation period of a few days, the initial symptoms of CCHF are a non-specific febrile illness that can occur suddenly. Sudden onset of fever, myalgia, diarrhea, nausea, and vomiting is typically reported. After this, patients enter the hemorrhagic period in which they begin exhibiting hemorrhages at various sites around the body ^8^. Case fatality rates can differ between outbreaks but typically range from 5% to 30% ^3^. However, subclinical or mild cases of CCHF may go unnoticed and may represent a substantial portion of CCHFV infections in humans ^9^. Despite the known genetic diversity of CCHFV, whether the infecting strain of CCHFV influences disease severity and outcome is unknown. High viral loads, absence of early antibody responses, and high levels of alanine aminotransferase (ALT) and aspartate aminotransferase (AST) are common predictors of poor outcome ^10– 14^ ( Figure 1). Thrombocytopenia and prolonged clotting times are also seen in severe cases ^12, 14, 15^. Levels of inflammatory cytokines are elevated in severe and fatal CCHF cases ^16– 19^, suggesting that CCHFV infection induces an inflammatory immune response.

The diagnosis of suspected CCHF cases can be accomplished by using reverse transcription–quantitative polymerase chain reaction (RT–qPCR) during the viremic phase of disease. RT–qPCR can also determine viral load, which often is correlated with disease outcome. An important consideration for these assays is the substantial genetic diversity of CCHFV; however, assays that can recognize a multitude of CCHFV genotypes have been developed ^20– 22^. Enzyme-linked immunosorbent assay and indirect immunofluorescent assay for the detection of human IgM and IgG CCHFV-specific antibodies are approaches of choice for serological diagnosis, and commercial kits are available. These tests may not be appropriate for suspected cases early during the acute phase of disease, as antibody responses are often absent or delayed in serious CCHF cases.

## Pathogenesis

Curiously, extensive serological evidence indicates that CCHFV can productively infect numerous vertebrate species, including both wild and domestic animals ^5^, yet humans seem to be uniquely susceptible to severe or lethal disease. Even within humans, CCHFV infection may result in a mild or subclinical infection ^9^, and why some patients develop a serious or fatal disease whereas others develop an asymptomatic infection is unknown.

The ability of CCHFV to cause severe or lethal disease in mice deficient in the type I interferon system but not wild-type (WT) mice ^23– 25^ suggests that innate immune responses in vertebrate hosts play a substantial role in limiting CCHFV pathogenesis. *In vitro* studies have identified retinoic acid-inducible gene I (RIG-I) as an innate immune sensor of CCHFV ^26^, although CCHFV may process its viral RNA to avoid RIG-I sensing ^27^. However, it is likely that more innate immune sensors contribute to sensing of CCHFV. Retrospective studies in humans have identified correlates between polymorphisms in Toll-like receptors (TLRs) and severity of disease, suggesting that TLRs may be an important immune-sensing pathway in the control of CCHFV. Polymorphisms in TLR7, 8, 9, and 10 have been found to be correlated with disease severity and outcome in infected patients in Turkey ^28– 30^. Polymorphisms in nuclear factor-kappa B have also been found to correlate with an increased risk of CCHF ^31^. Whether similar polymorphisms are associated with CCHF and case severity in populations in other geographic regions remains to be determined. Host apoptotic pathways may restrict CCHFV replication, as CCHFV replication can induce apoptosis ^32, 33^, and caspase 3, activated during apoptosis, can cleave the CCHFV nucleoprotein and inhibit viral replication ^34, 35^.

CCHFV also antagonizes innate immune signaling. The L segment of CCHFV encodes an ovarian tumor-like deubiquitinase (OTU) domain that recently has been shown to suppress innate immune responses likely by deubiquitinating proteins involved in innate immune signaling pathways ^36^. The CCHFV OTU domain can also cleave interferon-stimulated gene 15 (ISG15) modifications ^37, 38^. As ISG15 modifications have been implicated in direct antiviral effects and as ISG15 itself has been found to regulate the host innate immune response to viral infection ^39^, the de-ISGylation activity of the CCHFV OTU domain may be important for viral pathogenesis. Recombinant CCHFV encoding an OTU deficient in deubiquitinase activity or deficient in both deubiquitinase and de-ISGylation activity had reduced viral growth kinetics compared with WT CCHFV in interferon-competent but not interferon-incompetent cells, demonstrating that the OTU domain is important in overcoming interferon responses ^36^. Intriguingly, the CCHFV OTU domain has reduced affinity for mouse ISG15 compared with human ISG15, suggesting that mouse ISG15 may be an important barrier to CCHFV infection of mice ^40, 41^. The CCHFV OTU domain also has higher affinity for human ISG15 than the OTU domains of related *Nairoviridae* members that are not associated with disease as severe as CCHF, suggesting that the OTU domain may be an important virulence factor for CCHFV ^40^.

The role for adaptive immune responses against CCHFV in human pathogenesis is less clear. Low-to-absent anti-CCHFV antibody responses have been found to correlate with severe disease and death ^12– 14^, and levels of antibody may serve as a predictor of disease outcome. However, whether antibody responses contribute to the control of primary CCHFV infection is unknown, and neutralizing antibody responses even in survivors are typically low ^13^. Epitope mapping using linear peptides and serum from CCHFV survivors in Turkey and South Africa identified antibody responses toward epitopes in the mucin-like domain, GP38, and Gn protein ^42^ regions that are not likely to result in neutralizing antibodies ^43^. However, non-neutralizing antibodies were protective against lethal CCHFV challenge ^43^, demonstrating that antibodies can be protective via mechanisms other than neutralization. Studies evaluating a modified vaccinia virus-based vaccine for CCHFV found protection to require both cellular and humoral responses, suggesting that T cells may be an important component of vaccine-mediated protection against CCHFV ^44, 45^. The role for T cells in controlling primary CCHFV infection is unclear. Levels of circulating CD3 ^+^CD8 ^+^ T cells in peripheral blood were found to positively correlate with fatal outcome ^46^, and human CCHF survivors have been shown to exhibit long-lived CD8 ^+^ T-cell responses to CCHFV ^47^. Human leukocyte antigen alleles have also been found to correlate with protection and susceptibility to CCHFV ^48^. A humanized mouse model of CCHFV demonstrated that T cells were activated following CCHFV infection and CD8 ^+^ T cells had elevated levels of perforin, a marker of cytolytic activity ^49^. However, further studies are needed to understand how T-cell responses contribute to resolution of the infection.

Understanding the role of the adaptive immune response in the control of primary CCHFV infection has been limited by the lack of suitable animal models. Type I interferon-deficient mice exhibit a rapid-onset lethal disease prior to the development of adaptive immune responses, limiting the suitability of this model for studying adaptive immune responses. However, studies in STAT1-deficient mice demonstrated early activation of CD4 ^+^ and CD8 ^+^ T cells, although these cells were depleted from the spleen by day 3 post-infection (PI) and mice succumbed prior to detectable antibody responses ^25^. In a recent study using mice treated with an interferon blockade antibody, mice deficient in adaptive immune responses, but not WT mice, supported persistent viral replication for at least 2 weeks PI, demonstrating that adaptive immune responses can control CCHFV in mice ^50^. This model also demonstrated that adaptive immune responses, such as cytolytic T-cell activity, were not necessary for hepatic injury following CCHFV infection ^50^. These data suggest that CCHFV is directly capable of causing liver damage independent of the host adaptive immune response’s attempts to control viral replication. Furthermore, the recent development of a cynomolgus macaque model of CCHF ^51^ may provide insight into the role of the adaptive immune response and disease outcome. In this model, neither antibody titers nor neutralizing activity of the antibodies correlated with disease outcome ^51^. However, studies evaluating the contribution of the T-cell response to disease outcome in this model are still needed.

## Animal models

For many decades after the discovery and isolation of CCHFV, intracerebrally inoculated neonatal mice were used to propagate and detect CCHFV, as many other commonly used laboratory animals showed no disease following inoculation ^52^. Currently, the standard mouse model uses mice deficient in type I or both type I and type II interferon responses ^23– 25^ ( Table 1). Interferon-deficient mice typically exhibit a sudden onset of severe disease followed by death within 4 days PI, and mice exhibit high levels of inflammatory cytokines and elevated liver enzymes at time of death, similar to human CCHFV infections ^23– 25, 53^. Our lab recently described, in contrast to the sudden onset and death of CCHFV-infected mice in previous models, a model in which type I interferon-deficient mice infected with a clinical isolate, strain Hoti, develop a progressively worsening disease with several days of overt clinical signs followed by death at around day 7 or 8 ^54^. Similarly, low-dose challenge of mice with the clinical isolate Turkey-Kelkit06 strain resulted in a protracted but ultimately fatal disease ^55^. However, clinical isolates are capable of causing rapidly fatal disease in these mice as demonstrated for the Afg-09 strain ^53^. These distinct disease progressions of different CCHFV strains in mice suggest that there are important virulence determinants within CCHFV that remain to be described. Furthermore, a mouse model using the interferon blockade antibody MAR1-5A3 has been developed, allowing transient blockade of interferon signaling in a variety of mouse genetic backgrounds ^50, 56^. This model also allows the vaccination of fully immunocompetent animals that are rendered interferon deficient only at the time of CCHFV challenge, which may improve immune responses to certain vaccines ^56^.

**Table 1.** Animal models of Crimean–Congo hemorrhagic fever virus (CCHFV).

Mice	Cynomolgus macaques
• Type I interferon-deficient mice • Either genetic knock out or antibody blockade • Develop viremia, inflammatory immune responses, liver failure and rapid-onset terminal disease • Multiple CCHFV strains can be used. • Valuable for studying therapeutic interventions against CCHFV • Limited for studying host immune responses to CCHFV owing to innate immune deficiencies and death prior to adaptive immune responses • Humanized mice • Mice engrafted with human CD34 ^+^ hematopoietic stem cells • Develop a neurological-type disease • Strain-specific virulence observed	• Adult, immunocompetent macaques infected with CCHFV strain Hoti • Exhibit a spectrum of disease outcomes from asymptomatic to severe, lethal disease • Develop viremia, inflammatory immune responses, elevated liver enzymes, and increased clotting times • Major sites of viral replication are liver and spleen • Valuable pre-clinical model for therapeutic interventions against CCHFV • Can be used to study host and viral determinants of disease outcome

Recently, a humanized mouse model was described for CCHFV ^49^. In this model, irradiated mice are engrafted with human CD34 ^+^ hematopoietic stem cells. This model identified strain-specific virulence with humanized mice infected with a Turkey strain of CCHFV succumbing 2 to 3 weeks PI, whereas mice infected with an Oman strain survived until the study endpoint ^49^. Interestingly, the mice euthanized for terminal disease following CCHFV Turkey infection were euthanized for neurological manifestations, and high viral loads were found in the brains of euthanized mice ^49^ ( Table 1).

Until recently, there existed no immunocompetent animal model for CCHF. However, our lab recently described a cynomolgus macaque model of CCHF in which cynomolgus macaques infected with a human clinical isolate of CCHFV, strain Hoti, recapitulate many aspects of human CCHF cases ^51^ ( Table 1). Infected macaques develop a spectrum of disease outcome similar to that of human CCHF cases from asymptomatic to severe or lethal infections. Infected animals developed an early viremia, elevated levels of inflammatory cytokines, thrombocytopenia, and elevated liver enzymes similar to those of human cases of CCHF. Histological analysis showed that CCHFV infection resulted mainly in pathological changes in the liver and spleen, and *in situ* hybridization demonstrated infection of hepatocytes, Kupffer cells, and endothelial cells of the liver and marginal zone lymphocytes of the spleen and lymph nodes. Although this model is not uniformly lethal, the spectrum of clinical outcome following CCHFV infection within this model provides an opportunity to identify the host and viral mechanisms that contribute to disease outcome.

The role of the tick reservoir in CCHFV pathogenesis in vertebrates is largely unknown ^57^. Current animal studies use needle-delivered mammalian-cell culture-grown CCHFV, which excludes any contribution of tick-derived factors in these models. Tick saliva is capable of modulating a variety of early host defenses ^58^ and may play a role in the early immune responses to CCHFV, as tick saliva inhibited the migration of antigen-presenting cells *in vitro* ^59^. A tick transmission model involving infected ticks feeding on mice has recently shown that CCHFV genetic diversity may be shaped by the tick reservoir rather than the vertebrate hosts ^60^.

## Vaccines

Currently, the only vaccine for CCHFV is an inactivated preparation of virus grown in neonatal mouse brains ^61, 62^; however, this vaccine is used only in Bulgaria and is not approved for use in other countries with at-risk populations. The scalability and safety concerns of this type of vaccine will likely prevent widespread deployment of this vaccine, and new vaccine platforms for CCHFV are needed. An international collaboration among CCHFV researchers has been started to develop CCHFV vaccines and bring them to the clinic ( http://www.cchfvaccine.eu/), and several vaccines have shown promise in pre-clinical trials. A modified vaccinia virus expressing the glycoproteins of CCHFV was shown to provide 100% protection to lethally challenged mice ^44^. DNA-based vaccination or virus-like particle vaccination has also been shown to confer protection against lethal CCHFV challenge ^56, 63^. Mice fed transgenic plants expressing the CCHFV glycoproteins developed antibodies against the glycoproteins ^64^, although the efficacy of this platform against CCHFV challenge was not tested. Interestingly, although subunit vaccines containing the glycoproteins are immunogenic in mice ^65, 66^, they may not induce protective immune responses to CCHFV challenge ^65^. A formalin-inactivated preparation of CCHFV was also found to be protective against CCHFV infection in mice ^55^.

In addition to the glycoproteins, the nucleoprotein of CCHFV, encoded by the S segment, has been targeted by vaccines either on its own ^67, 68^ or in combination platforms that include both the nucleoprotein and the glycoproteins ^63^. Notably, vaccination with an adenovirus expressing the nucleoprotein of CCHFV provided substantial protection against lethal CCHFV challenge in mice ^67^, demonstrating that immune responses directed against the nucleoprotein can be protective independently of responses to the glycoproteins. However, a modified vaccinia virus expressing the nucleoprotein of CCHFV, though immunogenic, failed to protect against lethal CCHFV challenge ^68^, suggesting that protection afforded by nucleoprotein-based vaccines may be incomplete. Nevertheless, the greater sequence conservation of the nucleoprotein, even among divergent CCHFV strains, suggests that the nucleoprotein may be worth including in vaccine preparations to generate broadly protective immune responses.

## Antivirals

In addition to vaccines, investigations of antivirals against CCHFV have been conducted. Ribavirin, a nucleoside analog, is suggested by the World Health Organization for the treatment of CCHFV. However, clinical data supporting the use of ribavirin to treat CCHF are inconsistent; some studies report benefits whereas others report no benefit, and meta-analyses of multiple studies suggest that the efficacy of ribavirin is poor or inconclusive ^69– 71^. Notably, a placebo-controlled study failed to identify a clinical benefit of ribavirin treatment in patients with CCHF ^72^. A recent meta-analysis demonstrated that ribavirin treatment needs to be started soon after symptom onset (<48 hours) to reduce odds of death ^73^. In the recent emergence of CCHFV in Spain, although treatment of an infected nurse with ribavirin had mutagenic effects on CCHFV *in vivo* and a reduction in viral titers was coincident with treatment start ^74^, ultimately ribavirin treatment was discontinued because of suspected hemolytic anemia ^75^, a potential complication of ribavirin treatment ^76, 77^. The inconsistent data on the clinical benefit of ribavirin for the treatment of CCHFV and the potential for adverse events with ribavirin treatment have caused significant debate in the field ^78– 81^. Ethical considerations of placebo-controlled studies will likely make further studies of this type difficult ^82^, preventing definitive conclusions on the efficacy of ribavirin in patients with CCHF.

Studies in mouse models have also shown inconsistent efficacy of ribavirin. Two studies have shown that even early treatment with ribavirin (<6 hours PI) was unable to prevent lethal disease following infection with two distinct clinical isolates of CCHFV ^53, 54^. However, another study showed that ribavirin could protect against lethal disease following CCHFV strain 10200 infection when administered early and that protection diminished when treatment was delayed or challenge dose was increased ^25^. However, results from our lab showed that although early ribavirin treatment extended the mean time to death, ribavirin was unable to prevent death in 10200-infected mice ^54^. The reason for the distinct outcomes in ribavirin-treated strain 10200-infected mice seen between our study ^54^ and that by Bente *et al*. ^25^ is unknown but could be due to differences in mouse strain (IFNAR ^−/−^ versus STAT1 ^−/−^), challenge dose, or the time treatment was started after infection. Cumulatively, data in humans and mice suggest that while ribavirin may have limited clinical benefit in patients with CCHF, treatment likely needs to be started early in the course of disease to have clinical benefit. This may prove difficult, as the early symptoms of CCHF are non-specific and can progress rapidly to severe, hemorrhagic manifestations ^8^ and therefore patients may not present to health-care providers until exhibiting the more serious symptoms of CCHF.

Favipiravir is approved in Japan for the treatment of influenza virus infections ^83^ but has shown promise against other highly pathogenic RNA viruses, including Ebola ^84^ and Lassa ^85, 86^. Two studies have evaluated favipiravir against CCHFV *in vivo*. In a study by Oestereich *et al*., favipiravir treatment was effective in suppressing viral replication and preventing mortality following CCHFV infection, even when treatment was started 48 hours PI ^53^. Similarly, work by our group has shown that favipiravir treatment could be delayed until 6 days PI, a time point at which mice were exhibiting advanced disease, including death, and still offer significant clinical benefit to CCHFV-infected mice ^54^. These data suggest that favipiravir may be an effective antiviral for the treatment of advanced CCHF. Furthermore, Oestereich *et al*. demonstrated that favipiravir and ribavirin could synergistically inhibit CCHFV *in vitro*, allowing lower doses of both drugs to be used *in vivo* with clinical efficacy, suggesting that combination therapies in humans may be effective in treating CCHF while reducing unwanted side effects ^53^. A similar approach has been used in Lassa fever cases ^87^. In addition, a high-throughput screen using recombinant CCHFV identified a compound, 2′-deoxy-2′-fluorocytidine, with inhibitory activity superior to that of favipiravir or ribavirin *in vitro* ^88^. *In vivo* animal studies will be needed to evaluate how this compound performs in animal models of CCHF. Lastly, monoclonal antibodies have shown efficacy against CCHFV *in vivo* ^43^, and several clones were shown to neutralize divergent CCHFV strains ^89^, suggesting that they may have promise for the treatment of CCHF.

## Summary

In conclusion, much remains to be understood about the pathogenesis of CCHFV. However, the toolset for studying CCHFV has been steadily improving in recent years with the development of mouse and non-human primate models to a reverse genetics system for CCHFV that will facilitate dissection of the host and viral determinants of CCHFV pathogenesis. These tools will also allow the development and evaluation of novel therapies that reduce or prevent CCHFV-induced morbidity and mortality. Hopefully, the collaboration of multiple institutions in countries around the world toward the development of vaccines against CCHFV will lead to safe and effective vaccines for CCHFV being deployed in populations at risk for CCHFV infection. Lastly, the role of the tick vector in transmission and pathogenesis needs more attention, as does the role of livestock and other animal species in maintaining and transmitting CCHFV. This could lead to more effective measures to block CCHFV transmission.
